# 

**Published:** 1842-03

**Authors:** 


### CONTENTS

OF NO. III.

### ORIGINAL COMMUNICATIONS.

### ESSAYS AND CASES.

Art. I.—Observations on the epidemic Yellow Fever of Natchez, and of the South-west. By John W. Monette, M. D., of Washington, Mississippi. - - 161
Art. II.—On the Impropriety of Mercurial Salivation. By Dr. Thomas Townsend, of Wheeling, Virginia. - 183
Art. III.—Uterine Hemorrhage, with expulsion of the Ovum, successfully treated. By Dr. J. H. Holmes, of Spring Ridge, Mississippi. - - - - 192

### SELECTIONS FROM AMERICAN AND FOREIGN JOURNALS.

Action of Spirits upon Habitual Drunkards. - - 195
Absence of the Uterus. - - - - - 198
Rotatory Movements of the Yolk in the Ovum of Mammalia, during its passage through the Fallopian tube. - 199
Composition of False Membranes. - - - - 200
Evolution of Tubercle. - - - - - 201
M. Raciborski on the Physiology of Menstruation. - 203
Physiology of Conception. - - - - - 204

Diagnosis of Inflammation of the grey, and the white matter of the Brain. - - - - - - 206
Clinical Remarks by Dr. Marshall Hall, on the Use of Setons. - - - - - - 207
Reduction of Luxations of the Thigh. - - - 210
Reduction of a Prolapsus Uteri after sixteen years' continuance. 211
Union of Fractured Bones. - - - - 211
Ergot. - - - - - - - 212
Feigned Hysteria. - - - - - 213
Poisoning by Vapors of Antimony. - - - 214
Beneficial Effects of Narcotism in some obstinate cases of Neuralgia. - - - - - - 215
Remedies against Relapse in Intermittent Fever. - - 215
Treatment of Scrofulous Affections by the preparations of Walnut Leaves. - - - - - - 215
Prevention of Tubercles. - - - - - 216
The use of Opium in Acute Internal Inflammations. - 216
Antidote to the Salts of Copper. - - - 221
New Process for the Detection of Copper, applicable to medico-legal analysis. - - - - - 223
Statistics of the Lateral Operation of Lithotomy at Naples. 224
Reduction of a Strangulated Hernia, apparently effected by Acupuncture. - - - - - - 225
Hemorrhage after Lithotomy stopped by Creosote. - - 225
The Conditions which favor an excessive Size of the Fœtus. 226
Medical Quarrels. - - - - - - - 226
Researches into the real Constitution of the Atmosphere - 227
On the Influence of the last Inundation on the Health and Population of Lyons. - - - - - 228
Treatment of Asphyxia from Drowning. - - - 230
Connection between Abundance of Food and Mortality. - 233
Copy Right in Lectures. - - - - - - 233

ORIGINAL INTELLIGENCE.

The Trembles and Milk Sickness. - - - 235
Arsenic and the Trembles. - - - - 236
Kentucky School for the Instruction of the Blind. - - 237
Ohio Lunatic Asylum. - - - - - 237
Kentucky Lunatic Asylum. - - - - 238
Ohio School for the Blind. - - - 240

